# Supplementary material for: RADPAC-PD: A tool to support healthcare professionals in timely identifying palliative care needs of people with Parkinson’s disease
Source: PLoS One. 2020 Apr 21;15(4):e0230611. doi: 10.1371/journal.pone.0230611 (PMC7173770; doi:10.1371/journal.pone.0230611)
Supplement: S1 Table — (DOC) [file pone.0230611.s001.doc]

| **S2 Table Researchers’ characteristics** | | | | |
| --- | --- | --- | --- | --- |
| Code | Initials | Gender | Age | Occupation and experience |
| I1 | H.L. | Female | 35 | PhD candidate, master degrees in Social Sciences. 5 years experience on multiple projects for PD, nursing care and 14 years experience in PD-patient care. |
| I2 | M.S. | Female | 29 | Master degrees in Psychology. Two years experience on multiple projects for PD and 5 years employed as coordinator at ParkinsonNet, a Dutch nationwide PD network |
| I3 | M.G. | Female | 51 | Senior researcher, assistant professor, PhD in Palliative  Care, over 18 years of experience on research projects in  palliative care, senior lecturer Qualitative Research, Nurse  (NP) |
| I4 | M.M. | Male | 50 | Associate professor in healthcare innovation, director of strategy Movement Disorders Centre of Expertise, Managing Director ParkinsonNet, 17 years of experience on multiple multidisciplinary research and innovation projects for PD |
