# Supplementary material for: RADPAC-PD: A tool to support healthcare professionals in timely identifying palliative care needs of people with Parkinson’s disease
Source: PLoS One. 2020 Apr 21;15(4):e0230611. doi: 10.1371/journal.pone.0230611 (PMC7173770; doi:10.1371/journal.pone.0230611)
Supplement: S2 Table — (DOC) [file pone.0230611.s002.doc]

**S4 Table Characteristics of participants**

|  | Individual interviews | Focus group interviews | Delphi |  |  |  |
| --- | --- | --- | --- | --- | --- | --- |
|  |  |  | Total panel | First round | Second round | Third  round |
| Number of participants  (response rate, %) | 10* | 29* | 56** | 51  (93%) | 47  (84%) | 49  (88%) |
| Gender, male (%) | 30% | 10% | 20% | 19% | 19% | 16% |
| Professional background, n (%)  General practitioner  Neurologist  Psychiatrist  Specialist elderly care  PD nurse specialist  Nurse practitioner  Palliative care specialist  Physiotherapist  Community nurse  Psychologist  Speech therapist  Dietician  Occupational therapist  Other | -  1 (10)  1 (10)  1 (10)  1 (10)  1 (10)  -  1 (10)  1 (10)  -  1 (10)  1 (10)  1 (10)  - | -  1 (3)  -  2 (7)  6 (20)  1 (3)  -  10 (34)  1 (3)  1 (3)  3 (10)  2 (7)  3 (10)  - | 5 (9)  6 (11)  1 (2)  7 (12)  7 (12)  4 (7)  2 (4)  4 (7)  2 (4)  2 (4)  4 (7)  4 (7)  4 (7)  4 (7) | 4 (8)  5 (10)  1 (2)  6 (11)  7 (13)  3 (6)  2 (4)  4 (8)  1 (2)  2 (4)  4 (8)  4 (8)  4 (8)  4 (8) | 3 (6)  6 (13)  1 (2)  5 (11)  6 (13)  4 (8)  1 (2)  4 (9)  1 (2)  2 (4)  4 (9)  4 (9)  3 (6)  3 (6) | 3 (6)  6 (13)  1 (2)  6 (12)  7 (15)  3 (6)  2 (4)  3 (6)  1 (2)  2 (4)  4 (8)  4 (8)  4 (8)  3 (6) |
| Age (years), n (%)  25-34  35-44  45-54  55-64  65+ | 1 (10)  4 (40)  1 (10)  4 (40)  - | 2 (7)  4 (14)  11 (38)  11 (38)  1 (3) | -  -  -  -  - | 3 (6)  10 (19)  23 (44)  16 (31)  - | 3 (6)  9 (19)  20 (43)  15 (32)  - | 3 (6)  8 (16)  23 (47)  15 (31)  - |
| Participants (%) with expertise in the field of:  Parkinson’s disease  Palliative care  Both | 6 (60)  -  4 (40) | 23 (79)  -  6 (21) | -  -  - | 34 (65)  6 (12)  12 (23) | 29 (62)  6 (13)  12 (25) | 34 (70)  9 (14)  8 (16) |

*healthcare professionals volunteered in response to a general mailing with an unclear denominator and therefore a response could not be calculated

** healthcare professionals met the inclusion criteria. Age and field of expertise was not gathered at inclusion.
