# Supplementary material for: RADPAC-PD: A tool to support healthcare professionals in timely identifying palliative care needs of people with Parkinson’s disease
Source: PLoS One. 2020 Apr 21;15(4):e0230611. doi: 10.1371/journal.pone.0230611 (PMC7173770; doi:10.1371/journal.pone.0230611)
Supplement: S3 Table — (DOC) [file pone.0230611.s003.doc]

| **S5 Table Final statements and ratings on advance care planning and palliative care** | | | | |
| --- | --- | --- | --- | --- |
| **Statements *‘that did achieve’* agreement (in round X)** | I do not know (n) | % agreement among all respondents | % agreement of respondents with PD expertise | % agreement of respondents with expertise in palliative care and both |
| General statements on Advance Care Planning |  |  |  |  |
| 1. The ACP process will ideally be paced as agreed on by the patient, family and professionals collectively. (round 2) |  | 98 | 100 | 94 |
| 1. ACP discussions can reduce a patient’s and family members’ anxiety and uncertainties. (round 2) |  | 96 | 97 | 94 |
| 1. The ACP process includes frequent review of the care plan with the patient and family members (at least once a year). (round 1) | 1 | 96 | 97 | 94 |
| 1. All professionals are responsible for noting that the time has come to discuss ACP with a patient. (round 1) |  | 96 | 94 | 100 |
| 1. Doctors (general practitioners, elderly care physicians, neurologists) should have acquired the competencies to address ACP. (round 2) |  | 89 | 86 | 94 |
| 1. ACP is not specifically an element of palliative care, but rather part of optimal care provision. (round 2) |  | 89 | 90 | 89 |
| 1. Professionals (medical, nursing, allied health professionals) should always put a patients’ need first in the decision making process, even if it conflicts with the wishes of family. (round 1) | 2 | 84 | 85 | 83 |
| 1. Professionals should take the initiative to address ACP with a patient when 2 or more indicators are present. (round 3) |  | 78 | 79 | 73 |
| 1. Any professional is entitled to introduce ACP, but the primary treating practitioner takes final responsibility for the implementation of ACP. (round 2) | 4 | 77 | 79 | 72 |
| General statements on palliative care |  |  |  |  |
| 1. Palliative care is multidimensional and pays attention to an individual’s wellbeing in the physical, psychical, social and spiritual domains. (round 1) |  | 100 | 100 | 100 |
| 1. All professionals are responsible for identifying onset of the palliative phase. (round 1) | 1 | 100 | 100 | 100 |
| 1. PD is a progressive and incurable disease. (round 2) |  | 98 | 97 | 100 |
| 1. Timely identification of a patient’s end-of-life phase is important, because palliative care can then be provided in accordance to the patient’s values and wishes, sickness and dying. (round 1) | 3 | 94 | 93 | 94 |
| 1. PD treatment should focus on suppressing symptoms. (round 2) |  | 89 | 90 | 89 |
| 1. People with PD can die from PD, or more indirectly, from the complications of PD. (round 2) |  | 87 | 86 | 89 |
| Final set of indicators (RADPAC-PD) |  |  |  |  |
| Set of indicators for the ultimate moment to initiate ACP. (round 3)  With regard to the patient, is there any indication of the following? (answer: yes/no/unknown)   - Signals or asks for ACP or end-of-life care - Loses hope or dreads the future - Frequent falls (resulting in a hip fracture, for example) - Dysphagia or a first aspiration pneumonia episode - Cognitive deficits and/or neuropsychiatric problems - An (first) unattended hospital admission |  | 82 | 82 | 73 |
| Set of indicators for the start of the actual palliative phase. (round 3)  With regard to the patient, is there any indication of the following? (answer: yes/no/unknown)   - Preferred goal of care moves towards maximization of comfort - a transition in care needs, for example recurrent hospital admissions, nursing home admission and/or an increase in ADL help - PD drug treatment less effective or increasingly complex regime of drug treatments - several specific PD-symptoms or complications such as a significant weight loss, recurrent infections, progressive dysphagia, neuropsychiatric problems and/or multiple falls |  | 94 | 91 | 100 |
