# Supplementary material for: RADPAC-PD: A tool to support healthcare professionals in timely identifying palliative care needs of people with Parkinson’s disease
Source: PLoS One. 2020 Apr 21;15(4):e0230611. doi: 10.1371/journal.pone.0230611 (PMC7173770; doi:10.1371/journal.pone.0230611)
Supplement: S1 Appendix — (DOC) [file pone.0230611.s004.doc]

S1 Appendix Interview guide

| **Main question:** *“What experiences and opinions do professionals have about palliative care for people with PD and their caregivers?”*  Theme 1: Defining palliative care   - How would you define palliative care ? - What do you think about the definition of palliative care from WHO*? (a card with the following WHO definition was presented: “Palliative care is an approach that improves the quality of life of patients and their families facing the problem associated with life-threatening illness, through the prevention and relief of suffering by means of early identification and impeccable assessment and treatment of pain and other problems, physical, psychosocial and spiritual.”)   Theme 2: Experiences with palliative care   - What are your experiences with palliative care in daily practice? - What is the relevance of palliative care for people with PD? - How do you feel about timely starting palliative care for PD? When do you think is appropriate?   Theme 3: Needs of people with PD and their caregivers   - What are the palliative care needs of people with PD? - What are the palliative care needs of family caregivers of people with PD?   Theme 4: Offering palliative care in PD   - What specific (palliative care) interventions do you offer to people with PD and their family caregivers? - What are barriers and facilitators in offering palliative care from your professional background? - What do you think could contribute to the improvement of palliative care in PD? - What do you think about Specialist Palliative Care services in PD management? |
| --- |

*Source: WHO definition of palliative care. 2015. <http://www.who.int/cancer/palliative/definition/en/> Accessed on 14 May 2015.
