# Supplementary material for: RADPAC-PD: A tool to support healthcare professionals in timely identifying palliative care needs of people with Parkinson’s disease
Source: PLoS One. 2020 Apr 21;15(4):e0230611. doi: 10.1371/journal.pone.0230611 (PMC7173770; doi:10.1371/journal.pone.0230611)
Supplement: S2 Appendix — (DOCX) [file pone.0230611.s005.docx]

|  | S6 Appendix statements and ratings from round 1 (n=51) | | | | |
| --- | --- | --- | --- | --- | --- |
|  |  | I do not know (n) | % agreement among all respondents | % agreement of respondents with PD expertise | % agreement of respondents with expertise in palliative care and both |
| General statements on Advance Care Planning | | | | | |
| 1 | The ACP process includes frequent review of the care plan with the patient and family members (at least once a year). | 1 | 96 | 97 | 94 |
| 2 | All professionals are responsible for noting that the time has come to discuss ACP with a patient. |  | 96 | 94 | 100 |
| 3 | Professionals (medical, nursing, allied health professionals) should always put a patients’ need first in the decision making process, even if it conflicts with the wishes of family. | 2 | 84 | 85 | 83 |
| 4 | Nurse specialists or nurse practitioners are sufficiently competent to engage in ACP discussions. | 5 | 69 | 70 | 67 |
| 5 | Informing a patient with PD and family members about the poor prognosis facilitates future decision making, because they will be better prepared. | 2 | 68 | 59 | 83 |
| 6 | Writing a living will is essential if a patient is at risk of incompetence. | 4 | 67 | 67 | 67 |
| 7 | The doctor should take the initiative to introduce and monitor the ACP process. | 3 | 57 | 49 | 72 |
| 8 | The ACP process is primarily paced by the patient’s and family members’ willingness to confront end–of-life issues. | 1 | 49 | 46 | 56 |
| 9 | Doctors are sufficiently competent to engage in ACP discussions. | 8 | 41 | 39 | 44 |
| 10 | Informing a patient with PD and family members about the prognosis at the time the diagnosis has just been made is bound to worry them more than they should. | 2 | 34 | 36 | 28 |
| 11 | The ACP process and discussions about future medical decisions should be initiated when a patient is diagnosed with PD. |  | 24 | 9 | 50 |
| General statements on palliative care | | | | | |
| 12 | Palliative care is multidimensional and pays attention to an individual’s wellbeing in the physical, psychical, social and spiritual domains. |  | 100 | 100 | 100 |
| 13 | All professionals are responsible for identifying onset of the palliative phase. | 1 | 100 | 100 | 100 |
| 14 | Timely identification of a patient’s end-of-life phase is important, because palliative care can then be provided in accordance to the patient’s values and wishes, sickness and dying. | 3 | 94 | 93 | 94 |
| 15 | PD is a disease you can die from. |  | 50 | 46 | 56 |
| Set of indicators (RADPAC-PD, part 1 the ultimate moment to initiate ACP) | | | | | |
| 16 | A reason to discuss ACP is if the patient (and/or loved one) asks for ACP and/or if the patient (and/or loved one) wants to talk about end-of-life care. |  | 78 | 82 | 72 |
| 17 | A reason to discuss ACP is if the patient (and/or loved one) indicates a loss of hope and/or if the patient (and/or loved one) fears the future. | 1 | 78 | 85 | 67 |
| 18 | An initial conversation about ACP after a form of (Parkinson's) dementia has been diagnosed takes place too late. | 2 | 77 | 73 | 83 |
| 19 | An initial conversation about ACP after the palliative phase has begun takes place too late. | 1 | 71 | 73 | 67 |
| 20 | A first unplanned hospitalisation related to the symptoms of Parkinson's disease is a sign of the need for ACP. | 2 | 69 | 58 | 89 |
| 21 | The presence (or the increasing burden) of one or more of the following problems, is an indication for the need of ACP.   - Frequent falls (example: hip fracture) - Presence of swallowing problems and a first aspiration pneumonia - Nycturia involving nocturnal restlessness - Moderate amnesia with disorientation and difficulty with complex problem management - Presence of pseudohallucinations   - Loss of disinterest in preferred activities. | 4 | 66 | 58 | 67 |
| 22 | A reason to discuss ACP is admission to a nursing home. | 1 | 51 | 52 | 50 |
| Set of indicators (RADPAC-PD, part 2 the start of the actual palliative phase.) | | | | | |
| 23 | A landmark for the start of the palliative phase is if the patient (or loved one) aims only to maximize comfort. |  | 82 | 88 | 72 |
| 24 | If there is a progressive decline in physical and/or cognitive function despite optimal treatment, the palliative phase starts. | 3 | 67 | 64 | 72 |
| 25 | The palliative phase starts when the patient (or loved one) chooses to reduce, stop or abandon (further) treatment. | 1 | 67 | 73 | 56 |
| 26 | When the answer to the surprise question is "no", this then is a landmark for the start of the palliative phase. | 4 | 66 | 58 | 67 |
| 27 | The palliative phase starts when there are progressive swallowing problems despite treatment from a speech therapist. | 1 | 47 | 39 | 61 |
| 28 | Recurrent infections (such as urinary tract infections or respiratory infections) are a sign for the start of the palliative phase. | 4 | 43 | 36 | 56 |
| 29 | If the initial dosing or adjustment of antiparkinsonian medication no longer has any benefit over the side effects, the palliative phase starts. | 3 | 39 | 33 | 50 |
| 30 | The palliative phase starts when the patient (or loved one) asks for palliative care. | 2 | 33 | 30 | 39 |
| 31 | The palliative phase starts if the patient repeatedly encounters unplanned hospital admissions. | 2 | 31 | 24 | 44 |
| 32 | The palliative phase starts if the patient is admitted to a nursing home. | 1 | 22 | 15 | 33 |
| 33 | If the patient has mood disorders, the start of the palliative phase should be considered. | 3 | 12 | 6 | 22 |
| 34 | The palliative phase starts when the informal caregiver is severely overworked and needs more help and support. | 1 | 8 | 3 | 17 |

|  | S6 Appendix statements and ratings from round 2 (n=47) | | | | |
| --- | --- | --- | --- | --- | --- |
|  |  | I do not know (n) | % agreement among all respondents | % agreement of respondents with PD expertise | % agreement of respondents with expertise in palliative care and both |
| General statements on Advance Care Planning | | | | | |
| 1 | The ACP process will ideally be paced as agreed on by the patient, family and professionals collectively. (shared decision making) |  | 98 | 100 | 94 |
| 2 | ACP discussions can reduce a patient’s and family members’ anxiety and uncertainties. |  | 96 | 97 | 94 |
| 3 | Doctors (general practitioners, elderly care physicians, neurologists) should have acquired the competencies to address ACP. |  | 89 | 86 | 94 |
| 4 | ACP is not specifically an element of palliative care, but rather part of optimal care provision. |  | 89 | 90 | 89 |
| 5 | The first conversation about ACP should take place prior to the palliative phase. |  | 77 | 79 | 72 |
| 6 | Any professional is entitled to introduce ACP, but the primary treating practitioner takes final responsibility for the implementation of ACP. | 4 | 77 | 79 | 72 |
| 7 | Informing a patient with PD and family members about the poor prognosis facilitates future decision making, because they will be better prepared. | 2 | 68 | 59 | 83 |
| 8 | The ACP process includes frequent review of the care plan with the patient and family members (at least twice a year). | 2 | 66 | 66 | 67 |
| 9 | The ACP process is often too slow if this is paced only by the patient and family. | 10 | 53 | 48 | 61 |
| 10 | The ACP process and discussions about future medical decisions should be initiated when a patient is ‘ready’ for it and preferably not later than 1 year after a PD diagnosis. | 4 | 43 | 28 | 67 |
| 11 | The ACP process and discussions about future medical decisions should be initiated when a patient is ‘ready’ for it, and preferably not later than 6 months after a PD diagnosis. | 3 | 13 | 10 | 17 |
| 12 | The ACP process and conversation about future medical decisions should be initiated when a patient is ‘ready’ for it, and preferably not later than 3 months after a PD diagnosis. | 3 | 9 | 7 | 11 |
| General statements on palliative care | | | | | |
| 13 | PD is a progressive and incurable disease. |  | 98 | 97 | 100 |
| 14 | PD treatment should focus on suppressing symptoms. |  | 89 | 90 | 89 |
| 15 | People with PD can die from PD, or more indirectly, from the complications of PD. |  | 87 | 86 | 89 |
| 16 | Admission to the nursing home can be a signal that the patient's goals are more focused on comfort and preservation of function than on life-prolonging treatment. | 2 | 66 | 59 | 78 |
| 17 | The doctor is responsible for taking the initiative for marking the palliative phase |  | 57 | 83 | 56 |
| 18 | The capabilities of the informal caregiver have an impact on the patient's functioning. Overburdening of the informal caregiver can be an indication that the focus needs to change from care to comfort. | 6 | 51 | 55 | 44 |
| Set of indicators (RADPAC-PD, part 1 the ultimate moment to initiate ACP) | | | | | |
|  | Healthcare professionals evaluated single indicators on a three-point scale |  | 1; No signal (%) | 2; A supportive signal (%) | 3; An individual (isolated) signal (%) |
| 19 | When a patient or family asks to discuss ACP or end-of-life care. |  | 2 | 19 | 79 |
| 20 | A first ACP discussion is preferably held before a nursing home admission. If this did not happen, an admission can be an indicator for ACP. |  | 4 | 30 | 66 |
| 21 | When a patient/family member loses hope or dreads the future. |  | 2 | 43 | 55 |
| 22 | Presence of cognitive deficits and difficulty with complex problem solving. |  | 9 | 45 | 47 |
| 23 | Frequent falls (resulting in a hip fracture, for example). |  | 2 | 60 | 38 |
| 24 | Dysphagia or a first aspiration pneumonia episode. |  | 2 | 60 | 38 |
| 25 | Presence of hallucinations with awareness of illness. |  | 21 | 53 | 26 |
| 26 | Apathy or lack of interest in preferred activities. |  | 19 | 60 | 21 |
| Set of indicators (RADPAC-PD, part 2 the start of the actual palliative phase.) | | | | | |
|  | Healthcare professionals evaluated single indicators on a three-point scale |  | 1; No signal (%) | 2; A supportive signal (%) | 3; An individual (isolated) signal (%) |
| 27 | Presence of co-morbidity; presence of co-morbidity that reduces life expectancy or considerably restricts functioning. |  | 0 | 43 | 57 |
| 28 | If the benefits of start or adjustment of dopamine antagonist medication or other antiparkinson medication do not weigh against the side effects and the patient does not qualify for advanced treatment (such as DBS, Apomorphine or Duodopa). |  | 9 | 36 | 55 |
| 29 | If a patient (or loved one) opts to reduce, withdraw or withhold further treatment. |  | 9 | 38 | 53 |
| 30 | If a patient’s (or family members’) goals are primarily focused on maximization of comfort. |  | 2 | 47 | 51 |
| 31 | A negative answer to the surprise question. |  | 19 | 34 | 47 |
| 32 | When deterioration of independent functioning necessitates admission to a nursing home. |  | 17 | 43 | 40 |
| 33 | Presence of moderate memory loss with disorientation and difficulty with complex problem solving and/or dementia. |  | 13 | 57 | 39 |
| 34 | Presence of progressive dysphagia despite treatment by a speech therapist. |  | 6 | 55 | 38 |
| 35 | **Presence of neuropsychiatric symptoms (such as depression, hallucinations with awareness of illness, psychoses, apathy, and/or impulse control disorder.** |  | 11 | 51 | 38 |
| 36 | Persistent underweight despite optimal feeding. |  | 4 | 64 | 32 |
| 37 | If a patient repeatedly undergoes unplanned hospital admissions. |  | 6 | 62 | 32 |
| 38 | Presence of recurrent infections (such as urinary tract infection, or pneumonia). |  | 9 | 60 | 32 |
| 39 | When the goal of improving the patient’s functioning cannot be reached. |  | 15 | 57 | 28 |
| 40 | When for ADL/IADL moderate- to high dependence on an informal caregiver or home care worker develops. |  | 17 | 55 | 28 |
| 41 | When the goal of improving the patient’s functioning cannot be reached. |  | 15 | 57 | 28 |
| 42 | Presence of speaking problems that impair communication with others. |  | 30 | 49 | 21 |
| 43 | Presence of frequent on-off periods. |  | 38 | 40 | 21 |
| 44 | Considerable weight loss in the past few months. |  | 15 | 68 | 17 |
| 45 | Frequent falls. |  | 17 | 66 | 17 |
| 46 | Presence of nocturnal restlessness due to nocturia. |  | 28 | 55 | 17 |
| 47 | Presence of sleep disturbance, such as insomnia, excessive daytime sleepiness (EDS), vivid dreams, restless legs syndrome (RLS)/periodic leg movements disorder (PLMD) --- REM sleep behavior disorder (RBD). |  | 49 | 38 | 13 |
| 48 | Presence of pain with moderate-to-high impact on daily functioning. |  | 28 | 60 | 13 |
| 49 | Presence of constipation with moderate-to-high impact on daily functioning. |  | 60 | 30 | 11 |
| 50 | Presence of fatigue with moderate-to-high impact on daily functioning. |  | 36 | 55 | 9 |
| 51 | Presence of urge incontinence with moderate-to-high impact on daily functioning. |  | 57 | 34 | 9 |
| 52 | Presence of orthostatic hypotension with moderate-to-high impact on daily functioning. |  | 62 | 30 | 9 |

|  | S6 Appendix statements and ratings from round 3 (n=49) | | | | |
| --- | --- | --- | --- | --- | --- |
|  |  | I do not know (n) | % agreement among all respondents | % agreement of respondents with PD expertise | % agreement of respondents with expertise in palliative care and both |
| General statements on Advance Care Planning | | | | | |
| 1 | To what extend do you agree with following indicator as valuable contribution to the set of indicators (RADPAC-PD, part 1) “the presence of neuropsychiatric symptoms” | 2 | 78 | 79 | 73 |
| 2 | Professionals should take the initiative to address ACP with a patient when 2 or more indicators are present. |  | 78 | 79 | 73 |
| 3 | Professionals should take the initiative to address ACP with a patient when 1 or more indicators are present. |  | 69 | 68 | 73 |
| 4 | The ACP process and discussions about future medical decisions should be initiated when a patient is ‘ready’ for it, and preferably not later than 5 years after a PD diagnosis. | 3 | 51 | 53 | 47 |
| 5 | The ACP process and discussions about future medical decisions should be initiated when a patient is ‘ready’ for it, and preferably not later than 2 years after a PD diagnosis. | 3 | 39 | 32 | 53 |
| General statements on palliative care | | | | | |
| 6 | To what extent do you agree with the following statement: The Surprise Question is a valuable addition to the RADPAC-PD | 2 | 63 | 65 | 60 |
| 7 | At least 2 of the 4 indicators of the RADPAC-PD should be present for a professional to identify onset of a patient’s palliative phase | 1 | 59 | 62 | 53 |
| 8 | At least 1 of the 4 indicators of the RADPAC-PD should be present for a professional to identify onset of a patient’s palliative phase | 1 | 55 | 53 | 60 |
| 9 | At least 3 of the 4 indicators of the RADPAC-PD should be present for a professional to identify onset of a patient’s palliative phase | 2 | 41 | 44 | 33 |
| Final set of indicators (RADPAC-PD) | | | | | |
| 10 | Set of indicators (RADPAC-PD, part 1)  With regard to the patient, is there any indication of the following? (answer: yes/no/unknown)   - Signals or asks to discuss ACP or end-of-life care - Loses hope or dreads the future - Frequent falls (resulting in a hip fracture, for example) - Dysphagia or a first aspiration pneumonia episode - Cognitive deficits and/or neuropsychiatric problems - An (first) unattended hospital admission |  | 82 | 82 | 73 |
| 11 | Set of indicators (RADPAC-PD, part 2)  With regard to the patient, is there any indication of the following? (answer: yes/no/unknown)   1. Preferred goal of care moves towards maximization of comfort 2. a transition in care needs, for example recurrent hospital admissions, nursing home admission and/or an increase in ADL help 3. PD drug treatment less effective or increasingly complex regime of drug treatments 4. several specific PD-symptoms or complications such as a significant weight loss, recurrent infections, progressive dysphagia, neuropsychiatric problems and/or multiple falls | 1 | 94 | 91 | 100 |
